# Biogenesis, functions and clinical significance of circRNAs in gastric cancer

**DOI:** 10.1186/s12943-019-1069-0

**Published:** 2019-09-13

**Authors:** Chan Shan, Yinfeng Zhang, Xiaodan Hao, Jinning Gao, Xinzhe Chen, Kun Wang

**Affiliations:** Institute for Translational Medicine, College of Medicine, Qingdao University, Qingdao, 266021 China

**Keywords:** Circular RNA, Gastric cancer, Biomarker, Therapeutic target

## Abstract

Gastric cancer (GC) is one of the most common malignant tumours in the world and has high morbidity and mortality. Circular RNAs (circRNAs) are a class of non-coding RNAs with covalently linked circular structures. In recent years, plentiful circRNAs have been discovered that participate in many biological processes, including the initiation and development of tumours. Increasing evidences suggest important biological functions of circRNAs, implying that circRNAs may serve as vital new biomarkers and targets for disease diagnosis and prognosis. Among these, circRNAs are tend to aberrantly expressed and are regarded as potential biomarkers in the carcinogenesis and progression of GC. This review systematically summarised the biogenesis, biological properties and functions of circRNAs, with a focus on their relationship with GC, as well as their probable clinical implications on GC. As our cognition of the relation between circRNAs and GC deepens, more molecular mechanisms of GC progression will be discovered, and new therapeutic strategies will be used for the prevention and treatment of GC.

## Background

Gastric cancer (GC) is a high-recurrence-rate malignancy oncoma, which is the third major cause of cancer-related death worldwide, second only to lung cancer and liver cancer [1, 2]. Data show that the incidence and mortality of GC in Asian countries are increasing year after year, especially in the countries of East Asia, such as China, Japan and Korea [3–6]. In the last few decades, great progress has been made in GC treatment, and many attempts have been made to find effective treatment strategies. However, the morbidity and mortality of GC remain high.

Gastric carcinogenesis is a multistage, slowly progressive and multifactorial pathology process. *H. pylori* infection, obesity, excessive ingestion of salt and nitrate, and blood group A have been shown to be associated with an increased risk of GC [1]. Besides, genetic mutations, epigenetic alterations and aberrant molecular signalling pathways are involved in the processes of gastric carcinogenesis, spread and metastasis [7]. Thus, it is pivotal to identify the molecular patterns of GC and its specific biomarkers to develop treatments targeted to the specific tumour behaviour.

Accumulating reports have confirmed that many non-coding RNAs (ncRNAs), such as microRNAs (miRNAs) and long non-coding RNAs (lncRNAs), are associated with the carcinogenesis process of GC, and can be applied to be biomarkers in early risk assessment, clinic treatment and survival evaluation [8–10]. Beyond that, the participation of circular RNAs (circRNAs) in GC has been investigated.

CircRNAs are a new type of non-coding RNAs. They were firstly discovered in the Sendai virus by electron microscopy in 1976 [11]. Subsequently, researchers pointed out the existence of circRNAs in the cytoplasm of eukaryotic cells [12], yeast mitochondrion [13] and transcripts of human cells [14]. In the decades following the 1970s, circRNAs were considered to be the results of a splicing error of pre-mRNA processing with low abundance [14, 15]. With the development of high-throughput screening technology and prediction technique, the detected amounts and types of circRNAs are increasing at a rapid rate.

In recent years, circRNAs have come to be regarded as a newly appreciated class of non-coding RNAs. Increasing evidences suggest that circRNAs are involved in the occurrence and development of various diseases, such as cardiovascular diseases [16], neurological dysfunction [17, 18] and cancers [19, 20]. Moreover, due to the stability and tissue specificity of circRNAs, they act as potential biomarkers in evaluating ageing in *Drosophila* [21] and detecting diseases from human body fluid [22, 23]. Beyond that, the role of circRNAs in the process of cancer initiation and progression has especially gathered prominence; they have been recognised in hepatocellular carcinoma [24], breast cancer [25], colorectal cancer [26], and so on. CircRNAs may be considered dependable diagnostic and therapeutic molecular biomarkers for cancers [27].

In this paper, we summarise the current knowledge about the biogenesis and roles of circRNAs in GC and the potential clinical enlightenment for GC therapy.

## Circular RNAs

### Biogenesis of circRNAs

**Fig. 1:**
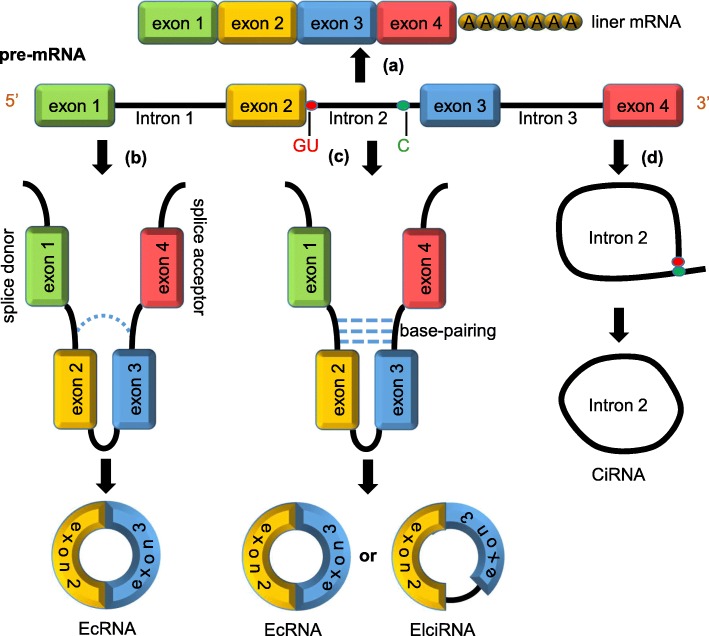
Biogenesis of circRNAs. **a** liner mRNA: a single-stranded ribonucleic acids carrying genetic information; (**b**) lariat-driven circularization: this model requires the covalently binding between the splicing donor and splicing acceptor, thus forming an exon-containing lariat; (**c**) intron-pairing-driven circularization: intronic complementary base-pairs bring the adjacent two exons close together. The exons and introns are then cut away by spliceosome to form cirRNAs; (**d**) intron cyclization: intron is cleaved from the pre-mRNA to form a ciRNA

CircRNAs are formed by different combinations of sequences and domains. According to the differences of origins, circRNAs can be divided into three groups: exonic circRNAs (ecRNAs) [28, 29], exon-intron circRNA (elciRNAs) [30] and circular intronic RNAs (ciRNAs) [31] (Fig. 1). CircRNAs were thought to be the result of exon-skipping events [32]. Two widely accepted models of circRNAs circularisation presented by Jeck et al. in 2013 are lariat-driven circularisation and intron-pairing-driven circularisation [33]. The mechanism of the two models are similar except for the first step. The former requires covalently binding between the splicing donor and splicing acceptor, thus forming an exon-containing lariat, which could then itself be internally spliced to an exon circle, thus forming ecRNAs [34] (Fig. 1b). In some cases, circRNAs stem from a single exon, whereas in others, the upstream exon attaches to the end of the downstream exon, thus forming circRNAs containing several exons [11]. Under some circumstances, if the intron between exons is retained, the cyclising transcript tends to form elciRNAs [30].

In the second cyclisation model, cyclisation happens because of intronic motifs bordering the circularised exon, which may bring the two exons close together through complementary base-pairs between introns (Fig. 1c).

Zhang et al. reported a new type of human circRNA stemming from introns that retain in the nucleus. The formation of intronic circRNAs depends on the 7 nt GU-rich element close to the 5′ splice site, and the 11 nt C-rich element near the branchpoint site [31]. During the process of backsplicing, the GU-rich element and the C-rich element first bind together to form a circular structure, then the exons and introns in this area are cut away by spliceosome (Fig. 1d). Additionally, circRNAs can also form from non-coding, intergenic genomic, antisense, 3′ UTR or 5′ UTR regions [11, 31].

The biogenesis of circRNAs is regulated by several kinds of factors, including intronic sequences, enzyme and protein factors. Zhang et al. demonstrated that exon cyclisation was dependent on the flanking intronic complementary sequences, thus making the circularisation process dynamic [28]. Moreover, exon circularisation efficiency can be regulated by competition between RNA pairing across flanking introns or within individual introns [35]. Adenosine deaminase acting on RNA (ADAR1) is a kind of RNA-editing enzyme. It has been reported that ADAR1 can negatively regulate the expression of circRNAs, whereas knocking down of ADAR1 induces elevated of circRNAs expression [36]. According to recent reports, RNA binding proteins (RBPs) might act as regulatory activators or inhibitors in the formation of circRNAs. Quaking (QKI) is an alternative splicing factor, which itself is up-regulated during human epithelial-mesenchymal transition (EMT). It has been reported that the formation of circRNAs was dependent on intronic QKI binding motifs. The overexpression of QKI is beneficial to the formation of circRNAs from normally linearly spliced transcripts [37]. Ashwal-Fluss et al. demonstrated that Muscleblind (MBL/MBNL1) could bind to its own second exon in pre-mRNA and determine its circularisation, thus competing against the canonical splicing, promoting the formation of circMBL while decreasing linear MBL level [35].

Briefly, the biogenesis of circRNAs and regulatory factors involved in circularisation remain unclear. More research is needed to help us understand these processes in depth.

### Biological properties of circRNAs

With the deepening of relevant research, many characteristics of circRNAs have been gradually verified. Among them, the following characteristics are recognised and important. (1) Diversity: circRNAs are widely and abundantly present in eukaryotic cells and have a wide variety [38]. In 2012, Salzman et al. showed that circRNAs could be produced from hundreds of human genes [39]; Jeck et al. detected 25,000 circRNAs in human fibroblasts [34]. (2) Highly abundant expression: the abundance of most circRNAs is lower than that of the linear transcripts, but in some cases, the expression of circRNAs is more abundant than the linear RNAs, even dozens of times moreso [33]. (3) Stability: unlike the linear RNAs with 5’ caps and 3’ poly A tails, circRNAs are characterised with single-stranded, covalently closed loop structures. Therefore, circRNAs are not easily degraded by Ribonuclease R (RNase R) and are more stable than linear RNAs [40]. (4) Conservation: most circRNAs are highly conserved among different species, and only a few circRNAs are not evolutionarily conserved [41]. Jeck et al. found that 2121 circRNAs found in human fibroblasts can be matched to the mouse genome [33]. In 2013, Memczak et al. identified 1950 human circRNAs and 1903 mouse circRNAs using RNA sequencing (RNA-seq) technology, of which 81 mouse circRNAs were identical to human circRNAs [11]. (5) Specificity: the expression of cireRNA is cell-type and spatial-temporal specific [42, 43].

### Functions of circRNAs

**Fig. 2:**
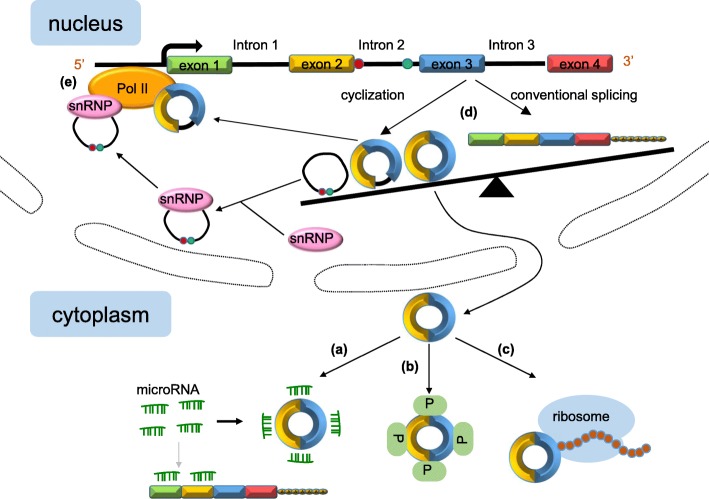
Functions of circRNAs. **a** circRNAs competitively bind miRNAs, so as to serve as intracellular competitive endogenous RNA, suppressing the effects of miRNAs on target genes. **b** circRNAs serve as protein baits or antagonists, thus arrestting the function of proteins, thereby affecting the related progresses. **c** circRNAs can translate to proteins through a cap-independent manner. **d** cirRNAs complete with the linear RNAs to get a balance. Some splicing factors may participate in this process, increasing the formation of circRNAs while reducing the linear splicing. **e** circRNAs interact with RNA polymerase II or transcription related factors, regulating the transcription and expression of parental genes

Distinguishing features reveal that circRNAs might possess vital functions (Fig. 2). Emerging evidence suggests that circRNAs participate in a series of pathophysiology processes.

#### CircRNAs serve as ceRNAs or miRNA sponges

MiRNAs are a class of endogenous, regulatory, non-coding RNAs found in eukaryotes that are about 20 to 25 nucleotides in length [8, 44]. Mature miRNAs are produced by the processing of a long primordial transcript through a series of nucleases, which are then assembled into an RNA-induced silencing complex (RISC) [11]. MiRNAs can recognise target mRNAs by means of base-complementary pairing, direct silencing RISC to degrade target mRNAs, or repress translation of target mRNAs [44]. CircRNA is widely distributed in eukaryotic cells and has multiple miRNA response elements (MER), which can competitively bind miRNAs and act as intracellular competitive endogenous RNA (ceRNA), removing the inhibitory effect of miRNAs on target genes, thus regulating miRNAs’ function and expression of related genes [45] (Fig. 2a).

The most studied miRNA in circRNA research is miR-7, which participates in the regulation of multiple biological events in disease and tumorigenesis, such as cellular proliferation, migration, invasion, differentiation and tumour cell signal transduction [46, 47]. MiR-7 has been determined as an oncogene as well as a tumour suppressor in a number of malignancies including breast [48], brain [49], head and neck [50], lung [51], liver [52] and melanoma cancer [53]. The recently identified circular RNA, ciRS-7 (also termed CDR1as), which acts as a designated miR-7 inhibitor/sponge, has conceptually changed the mechanistic understanding of miRNA networks [45]. As a circular miR-7 inhibitor, ciRS-7 harbours more than 70 conventional miR-7 binding sites [54]. Expression of ciRS-7 efficiently recruits miR-7, resulting in decreased miR-7 function and up-regulation of miR-7 target genes. In the mouse brain, in situ profiling of miR-7 and ciRS-7 showed a remarkable overlap in expression, and it is likely that the vast majority of especially brain-expressed miR-7 is tethered to ciRS-7 [45]. In oesophageal squamous cell carcinoma, ciRS-7 promoted growth and metastasis via regulation of miR-7/HOXB13 [55]. This is consistent with the miRNA sponge and ceRNA hypothesis.

The miRNA sponge may be a universal biological function of circRNAs. The sex determining region Y (SRY) gene consists of only one exon. In the early stage of development, its transcript takes the form of linear RNA molecules, which, in turn, guide protein translation. However, in adult testes, SRY is mainly present in the cytoplasm in a cyclic form and does not have a translation function [56]. Further studies revealed that the circular transcript of the SRY gene, which contains 16 MREs of miR-138, acting as a miR-138 sponge, inhibited the activity of miR-138 and regulated the expression level of miR-138 target genes [45].

#### CircRNAs bind with proteins

Serving as protein baits or antagonists is another important role of circRNAs (Fig. 2b). For example, circ-Foxo3 is down-regulated in cancer cells and is related to apoptosis and cell proliferation [57]. In mouse cancer cells, circ-Foxo3 represses cell cycle progression by the interaction with cyclin-dependent kinase inhibitor 1 (p21) and cyclin-dependent kinase 2 (CDK2), thereby blocking the roles of p21 and CDK2 in cell cycle regulation [19]. These studies demonstrate that circRNAs might act as decoys to influence the function of proteins within cells.

#### Potential translation templates of proteins

Most circRNAs are present in the cytosol, suggesting that they may be assembled into ribosomes for translating into proteins (Fig. 2c). Unlike many linear mRNAs with a 5’ cap and a 3’ poly A tail, circRNAs lack efficient translation initiation structures, but can be translated once an internal ribosome entry site (IRES) is initiated [58]. Chen et al. found that eukaryotic ribosomes could initiate translation after inserting an IRES into a synthetic circRNA, which indicates that the 5’ end of mRNA is not necessarily the entry for ribosomal 40S subunit [59].

In addition, a circRNA containing a green fluorescent protein open reading frame produces long protein chains in *Escherichia coli* [60]*.* The core of the hepatitis D virus contains a single-stranded negative-chain covalently closed circRNA, which encodes a hepatitis D virus antigen (HDAg) that plays an important role in disease progression [61]. Moreover, it was reported that circ-ZNF609 was associated with the ribosome and could be translated into a protein, thus controlling myoblast proliferation [62]. Importantly, researchers found that circMbl could produce a protein in fly heads [63]. Additionally, Yang et al. demonstrated that N-methyladensine (m^6^A) could promote the initiation of protein translation from circRNAs in human cells [64]. The m^6^A recognition protein YTHDF3 binds to the modification site of circRNAs and recruits eIF4G2 and other translation initiation factors to drive the translation of circRNAs [65].

#### CircRNAs regulate the splicing of pre-RNA

CircRNAs can not only directly bind to proteins but also indirectly relate to proteins through RNA-mediated interaction, which affects alternative splicing, thereby regulating gene expression (Fig. 2d). Ashwal-Fluss and others found that circMbl was generated from the second exon of the splicing factor MBL, which competed with conventional pre-mRNA splicing. Interestingly, MBL could promote the formation of circMbl and, at the same time, closely bind to circMbl, thereby decreasing the effective concentration of MBL and reducing the formation of circMbl [35]. In addition, once increasing the linear splicing in *Drosophila* and human cells, the amount of circRNA production will reduce accordingly, suggesting that the cyclisation of pre-mRNA may compete with its linear splicing to achieve a balance between circularisation and linear splicing status. A circRNA from the *SEPALLATA3* gene regulates splicing of its cognate mRNA through R-loop formation, causing the suspension of gene transcription, at the same time, promoting the recruitment of splicing factors to the transcripts, and affecting the alternative splicing [66].

#### Regulation of parental gene expression by circRNAs

ElciRNAs and ciRNAs contain few relatively dispersed miRNA binding sites, causing a lack of the miRNA sponge effect. Recent research has demonstrated that elciRNAs and ciRNAs have the function of regulating RNA polymerase II (Pol II) transcript activity, thereby managing the expression of parental genes (Fig. 2e). Particularly, knocking down of ci-ankrd52 and ci-sirt7 leads to a significant reduction of the transcription of their parental genes by associating with the elongation Pol II complex [31]. Liu et al. pointed out that when localising in the nucleus, elciRNAs (for example, circEIF3J and circPAIP2) might associate with U1 small nuclear ribonucleic proteins (snRNPs) through RNA-RNA interaction [67]. Then the elciRNA-U1 snRNP complexes might further interact with the Pol II transcription complex at the promoters of parental genes to enhance gene expression [30]. In addition, circRNA cANRIL may modulate the amount of ANRIL protein and regulate the expression of its parental gene INK4/ARF [68]. In brief, circRNAs might regulate parental gene expression by interacting with Pol II as well as other transcription-related factors.

In addition, it worth noting that there are some elements, such as cell types, subcellular localization and abundance of circRNAs, objects that interact with circRNAs, upstream regulators and downstream targets of circRNAs, relative signalling pathways, form the so-called “cellular context” that influences the final activities of circRNAs [69]. For example, circFAT1 was up-regulated in osteosarcoma (OS) [70]. Liu et al. reported that circFAT1 regulated Yes-associated protein 1 expression by sponging miR-375 in cytoplasm, thereby promoting the proliferation, invasion and metastasis in OS cells. Whereas, in GC cells, circFAT1 was downregulated and was distributed in cytoplasm as well as in nucleus [71]. Mechanismally, circFAT1 inhibited the proliferation, metastasis and invasion of GC cells through miR-548 g/RUNX1 axis in cytoplasm. Conflicting reports also emerged in circHIPK3 functions. CircHIPK3 was downregulated in bladder cancer and suppressed tumor growth, angiogenesis and metastasis by sponging miR-558 [72]. While in GC tissues and cells, circHIPK3 was up-regulated and acted as the sponge of miR-124 and miR-29b, thus promoting the proliferation process of GC cells [73]. In fact, cellular context-dependent circRNAs functioning might be a feature of circRNA-mediated regulation. Under the cellular and overall context in cancer, circRNAs might serve as a much complex regulator.

## CircRNAs and GC

Numerous studies have shown that circRNAs are aberrantly expressed in many cancer tissues. In the last few decades, quantity of circRNAs have been found in tumorous tissues, including in GC, indicating that circRNA could be utilized in diagnostic and therapeutic applications [74].

With the deepening of the research on the pathogenesis of GC, many differentially expressed circRNAs in GC tissues and cells have been found. These differentially expressed circRNAs eventually lead to changes in biological, genetic information. Increasing evidences prove that circRNAs play an important role in the development and progression of GC. Here we summarized the dysregulated circRNAs that have been found related with GC (Table 1).

**Table 1:** Dysregulated circRNAs in GC

Name	Dysregulation	Sponge target	Function	Types of GC tissues and GC cell lines	Ref.
circPVT1	up-regulated	miR-125 family	Independent prognostic indicator; promotes cancer cell proliferation	187 GC tissue samples with various clinicopathologic features; MGC-803 and AGS GC cell lines	[75]
circRNA_100269	downregulated	miR-630	Inhibits cancer cell proliferation	112 GC tissue samples with various clinicopathologic features; AGS, MKN-28, MKN-45, BGC-823, MGC-803 and SGC-7901 GC cell lines	[76]
circNRIP1	up-regulated	miR-149-5p	Promotes cancer cell proliferation, invasion and migration	80 GC tissue samples with various clinicopathologic features; BGC-823, AGS, SGC-7901, MGC-803, MKN-45 and HGC-27 GC cell lines	[77]
circ-DONSON	up-regulated	–	Promotes cancer cell proliferation, invasion and migration	142 GC tissue samples; BGC-823, AGS, MGC-803, MKN74, HGC-27 and SGC-7901 GC cell lines	[78]
circRNA0047905	up-regulated	miR4516 miR1227-5p	Tumor promoter; potential therapy target	31 GC tissue samples; AGS GC cell line	[79]
circDLST	up-regulated	miR-502-5p	Promotes cancer cell proliferation, invasion and metastasis	396 GC tissue samples of various stages; MGC-803, BGC-823, SGC-7901, HGC-27, AGS, MKN-45 and MKN-28 GC cell lines	[80]
circ_0067997	up-regulated	miR-515-5p	Promotes cancer cell viability, proliferation and invasion	48 GC tissue samples with various clinicopathologic features; SGC-7901, MGC-803, BGC-823 and MKN28 GC cell lines	[81]
circCACTIN	up-regulated	miR-331-3p	Promotes GC cells migration, invasion and EMT	32 gastric adenocarcinoma tissue samples; GES1, BGC-823, MGC-803 and SGC-7901 GC cell lines	[82]
ciRS-7	up-regulated	miR-7	Promotes cancer cell proliferation	256 GC tissue samples (102 in training cohort and 154 in validation cohort) with various clinicopathologic features; MGC-803 and HGC-27 GC cell lines	[83]
circHIPK3	up-regulated	miR-124 miR-29b	Promotes cancer cell proliferation	63 GC tissue samples (28 infiltrative type samples and 35 expanding type samples); XGC-1 (infiltrative type) and XGC-2 (expanding type) GC cell lines	[73]
circPDSS1	up-regulated	miR-186-5p	Promotes cell cycle and proliferation, inhibits cell apoptosis	20 GC tissue samples; MGC-803, HGC-27, and BGC-823 GC cell lines	[84]
circFAT1	downregulated	miR-548 g	Inhibits cancer cell proliferation, invasion and migration	38 GC tissue samples with various clinicopathologic features; AGS,SGC-7901, BGC-823, MKN-28, MGC-803 and MKN-45 GC cell lines	[71]
circNF1	up-regulated	miR-16	Promotes cancer cell proliferation	23 GC tissue samples with various clinicopathologic features; MKN-28, NCI-N87, AGS, KATOIII, RF1, RF-48 GC cell lines	[85]
circYAP1	downregulated	miR-367-5p	Tumor suppressor, inhibits cell growth and invasion	80 GC tissue samples of various stages (13 of stage I, 25 of stage II, 38 with stage III and 4 of stage IV); HGC-27 GC cell line	[86]
hsa_circ_0000993	downregulated	miR-214-5p	Inhibits cellular migration, invasion and proliferation	Male patients tissue samples with stage III A primary GC (aged 59, 67 and 69 years, respectively); SGC-7901 and BGC-823 GC cell lines	[87]
circ-ZFR	downregulated	miR-107 miR-130a	Inhibits GC cell propagation, cell cycle and promotes apoptosis	48 GC tissue samples; AGS, AZ521, and HGC-27 GC cell lines	[88]
circHECTD1	up-regulated	miR-1256	Promotes glutaminolysis, proliferation, migration, and invasion of cancer cells; promotes autophagy; potential therapeutic target	GC tissue samples with various clinicopathologic features; BGC-823, MKN-45, HGC-27, AGS, MGC-803, and SGC-7901 GC cell lines	[89]
ciRS-133	up-regulated	miR-133	Aggravates tumour cachexia	Tissue and plasma samples of GC patients; SGC-7901 GC cell line	[90]
circAKT3	up-regulated*	miR-198	Enhances resistance to Cisplatin chemotherapy; therapeutic target	149 GC tissue samples (patients received CDDP treatment with/ without tumor relapse); SGC-7901 and BGC-823 GC cell lines and their CDDP-resistant strains SGC-7901CDDP and BGC-823CDDP	[91]

*CircAKT3 is up-regulated in CDDP-resistant GC samples compared to CDDP-sensitive samples

### Expression of circRNAs in GC

With the development of high-throughput sequencing, biochip technology and bioinformatics, more and more circRNAs have been found unconventionally expressed in GC and may have potential functions. Chen et al. distinguished 180 differently expressed circRNAs by RNA-seq analysis, among which 82 were up-regulated, and 98 were downregulated in GC tissues compared with normal tissues [75]. Interestingly, approximately 80% of these circRNAs were produced from protein coding genes. Shao et al. reported a total of 308 circRNAs, among which 34.7% (107) were up-regulated and 65.3% (201) were downregulated in GC tissues [92]. Dang et al. performed a microarray screening analysis and confirmed 713 differently expressed circRNAs between GC and non-GC tissues, including 191 up-regulated circRNAs, and 522 downregulated circRNAs [93]. Lai et al. revealed 204 differently expressed circRNAs in five paired GC tissues and adjacent normal tissues; 71 of these circRNAs were up-regulated, and 133 were downregulated in GC tissues [94]. Zhang et al. found 95 circRNAs up-regulated (> 4 folds), and 94 circRNAs downregulated (< 0.25 folds) in the GC tissues [90]. In particular, hsa_circ_0010522 showed the highest change folds in the GC group compared to the normal group. In another study, Fang et al. acquired 250 differently expressed circRNAs in the GC group from the GEO database [71]. Rong et al. detected 3443 significantly altered circRNAs in GC samples with a lymph node metastasis group and no lymph node metastasis group; GO analysis revealed that these circRNAs were related to critical molecular functions and several physiological processes [95].

### CircRNAs and the proliferation and progression of GC

Abnormal proliferation and invasiveness of cells are characteristic changes in tumour cells. The proliferation and invasion of GC cells is a complex process involving multiple factors, genes and steps. At present, the mechanism of these processes remains ambiguous.

Chen et al. characterised circPVT1 from 5500 differently expressed circRNAs in GC tissues compared with relevant normal tissues [75]. Further investigation revealed that circPVT1 was up-regulated in GC tissues and cell lines, and functioned as a promoter of cell proliferation due to the role of the sponge of the miR-125 family. CircPVT1 might serve as a proliferation and progression factor in GC. Zhang and colleagues studied the interaction and function of circRNA_100269 and its downstream target miRNAs [76]. They found that circRNA_100269 and the linear mRNA LPHA2 were downregulated in GC tissues and further suppress cellular growth. Through bioinformatics prediction and experimental verification, they confirmed miR-630 as the direct target of circRNA_100269. MiR-630 mimics suppressed the function of circRNA_100269, thus promoting the proliferation and progression of GC cells. The study of Zhang et al. revealed that circLARP4, a circRNA produced from the exon 9 and 10 of LARP4, was downregulated in GC tissues and cells; overexpression of circLARP4 caused the suppression of cell growth and invasion [96]. Furthermore, they pointed out that circLARP4 could act as the sponge of miR-424; overexpression of circLARP4 weakened the proliferative effect by miR-424 in a Yap-dependent signalling pathway in several kinds of GC cell lines.

Additionally, Zhang’s group demonstrated that circNRIP1, a circRNA raised from the NRIP gene, was correlated with the tumour size of GC and lymphatic invasion [77]. Further investigation verified that circNPIP1 could directly interact with and serve as the sponge of miR-149-5p, thus regulating the downstream AKT1/mTOR pathway in GC tissues and cell lines. Ding et al. discovered that circ-DONSON was up-regulated in GC cells [78]. Knocking down of circ-DONSON inhibited proliferation, invasion and migration of GC cells significantly, while at the same time, promoted apoptosis. Conversely, overexpression of SOX4 facilitated the proliferation of GC cells in a Wnt signalling pathway-dependent manner.

Moreover, circRNA0047905 was a tumor promoter in GC that directly binds to miR-4516 and miR-1227-5p, thereby alleviating the inhibition of the downstream targets MMP11 and SERPINB5 in GC cell lines [79]. Zhan et al. demonstrated that circDLST could act as the sponge of miR-502-5p, further regulate the NRAS/MEK1/ERK1/2 signalling in GC cells [80]. Knocking down of circDLST suppressed cellular viability, invasion and metastasis of GC in vitro and in vivo. In a new study of Zhang’s group, they found that circ_0067997 was an oncogene in GC, which acts as miR-515-5p sponge, thereby regulating the downstream gene XIAP to promote cellular proliferation and invasion [81]. Zhang et al. observed that circCACTIN was beneficial to GC cell invasion, migration as well as EMT [82]. Mechanismally, circCACTIN affected GC progression by serving as miR-331-3p sponge and regulating transforming growth factor-β receptor type 1 expression. These studies provided an approach that circRNAs could sequester specific miRNAs, thereby regulate target genes and signalling pathways associated with proliferation, differentiation, invasion, and migration of GC cells.

### CircRNAs as diagnostic and prognostic biomarkers in GC

Although vast majority of patients with early GC can be cured by surgery, most patients are diagnosed at the advanced stage of cancer and lose the best opportunity for surgical treatment due to the lack of reliable and effective early diagnosis techniques. Endoscopy is currently the gold standard for the diagnosis of GC, but this invasive technique is extremely painful for the patient, and the results depend on the skills and experiences of the doctors. With the ongoing development of sequencing technology and biotechnology, disease-related RNAs have been found in human saliva, gastric juice, plasma and other human components, and some have been used as diagnostic markers [97, 98]. These findings suggest that circRNA has great potential as a novel biomarker and provides new ideas for early diagnosis. Additionally, prognosis evaluation plays an important role in early intervention of poor prognostic factors and prolonging the lifespan of patients. Recent studies have shown that circRNAs are involved in the pathological process of GC. Increasing studies have identified circRNAs as diagnosis and prognosis biomarkers of GC. CircRNAs that might serve as diagnostic and prognostic biomarkers are listed in Table 2. Those circRNAs with an area under the curve (AUC) > 0.75 are detailed.

**Table 2:** CircRNAs as diagnostic and prognostic biomarkers in GC

Name	Dysregulation	Function	Sensitivity	Specificity	Cut-off value (ΔCt)	AUC	Clinicopathological association	Ref.
circPVT1	up-regulated	Independent prognostic indicator;	–	–	–	0.605	Tumor stage and overall survival time	[75]
hsa_circ_0014717	downregulated	Diagnostic biomarker	59.38%	81.25%	12.14	0.696	Tumor stage, distal metastasis, CEA, and CA19–9	[92]
circLARP4	downregulated	Independent prognostic indicator	67.4%	67.6%	20.37	0.64	Tumor size and lymphatic metastasis	[96]
circ-KIAA1244	downregulated	Independent prognostic indicator	77.42%	68.00%	1.443	0.748	TNM stage, lymphatic metastasis and overall survival time	[99]
hsa_circ_0000467	up-regulated	Independent prognostic factor; potential target	70.5%	64.8%	–	0.799	Lymphatic invasion, and TNM stage	[100]
hsa_circ_0000190	downregulated	Diagnosis biomarker	72.1%	68.3%	6.83	0.75	Tumor size, lymphatic metastasis, distal metastasis, TNM stage and CA19–9	[101]
hsa_circ_002059	downregulated	Diagnosis biomarker	81%	62%	12.9	0.73	TNM stage, distal metastasis, gender and age	[102]
hsa_circ_0000181	downregulated	Diagnosis biomarker	20.6%	99%	7.27	0.582	Differentiation and CEA (plasma)	[103]
			85.2%	53.9%	9.4	0.756	Tumor diameter, lymphatic metastasis, distal metastasis and CA19–9 (tissue)	
hsa_circ_0000096	downregulated	Diagnosis biomarker	–	–	12.9	0.82	Gender, invasion and TNM stage	[104]
hsa_circ_0003159	downregulated	Diagnosis biomarker	85.2%	56.5%	12.31	0.75	Gender, TNM stage and distal metastasis	[105]
circPSMC3	downregulated	Diagnosis and prognosis biomarker	85.85%	95.24%	9.965	0.933	TNM stage and lymphatic metastasis	[95]
hsa_circ_0000745	downregulated	Diagnosis biomarker	85.5%	45%	–	0.683	TNM stage (plasma) and tumor differentiation (tissue)	[106]
hsa_circ_0006633	downregulated	Diagnosis biomarker	60%	81%	8.17	0.741	Distal metastasis and CEA	[107]
hsa_circ_0001649	downregulated	Independent prognostic indicator and diagnosis biomarker	71.1%	81.6%	0.227	0.834	Tumor differentiation	[108]
hsa_circ_0001895	downregulated	Prognosis biomarker	67.8%	85.7%	9.53	0.792	Cell differentiation, Borrmann type, and CEA	[109]
has_circ_0074362	downregulated	Diagnosis biomarker	84.3%	36.2%	12.17	0.63	Lymphatic metastasis and CA19–9	[110]
hsa_circ_102958	up-regulated	Diagnosis biomarker	61%	86%	–	0.74	TNM stage	[111]
has_circ_0066444	up-regulated	Diagnosis biomarker	70.75%	68.87%	–	0.733	Lymph metastasis	[112]

*AUC* Area under the curve, *CA19–9* Carbohydrate antigen 19–9, *CEA* Carcinoembryonic antigen

Tang’s study revealed that circ-KIAA1244 was downregulated in GC tissues, plasmas and cells compared with the normal control [99]. Clinical analysis showed that low expression of circ-KIAA1244 was associated with TNM stage, lymphatic metastasis and shorter overall survival time in GC patients. Circ-KIAA1244 could be used as a new biomarker for the diagnosis and prognosis of GC. Besides, the expression level of hsa_circ_0000467 was significantly high in GC tissues, plasmas and GC cells than the normal control, and was associated with TNM stage and lymph metastasis of GC [100]. The AUC of hsa_circ_0000467 was 0.799, which was preeminent than some biomarkers already exist, like carcinoembryonic antigen (CEA). Cytobiological studies showed that silencing of hsa_circ_0000467 inhibited proliferation, migration, and invasion of GC cells. Hsa_circ_0000467 might become a biomarker for the diagnosis and prognosis as well as a potential therapeutic target of GC. Furthermore, hsa_circ_0000190 was proved to be downregulated in tissues and plasmas of GC patients [101]. The expression of hsa_circ_0000190 was correlated with tumor size, lymphatic metastasis, distal metastasis, TNM stage and carbohydrate antigen 19–9 (CA19–9) levels. Hsa_circ_0000190 can be regarded as a novel diagnosis biomarker of GC that better than CEA and CA19–9.

Additionally, Zhao’s group found that hsa_circ_0000181 was significantly downregulated in GC tissue and plasma samples than the adjacent non-tumorous samples and healthy group [103]. Clinical study showed that hsa_circ_0000181 level in plasmas of GC patients was connected with differentiation and CEA level, whereas its expression in GC tissues was associated with tumor diameter, lymphatic metastasis, distal metastasis and CA19–9 level. Plasma and tissue hsa_circ_0000181 might be a stable diagnosis marker of GC. Hsa_circ_0000096 has been shown to be downregulated in GC tissues and GC cell lines compared with the adjacent tissues and normal gastric epithelial cells [104]. The expression level of hsa_circ_0000096 was associated with some clinicopathological features of GC, like patients’ gender, invasion and TNM stage. Moreover, a downregulated circRNA in GC tissues, hsa_circ_0003159, has been reported to be negatively associated with gender, distal metastasis, and tumor stage [105]. The AUC of hsa_circ_0003159 is 0.75, and the sensitivity and specificity are 85.2 and 56.5%, respectively.

In a recent research of Rong et al., they reported that circPSMC3 possessed a decreased level in GC tissue and plasma samples and GC cell lines compared to normal control [95]. Lower expression of circPSMC3 was negatively correlated with TNM stage and lymphatic metastasis. Importantly, circPSMC3 showed a fairly high AUC of 0.933, which represents a remarkably reliable diagnostic value. Hsa_circ_0001649 was a well-known circRNA that acted as a diagnostic marker and tumor suppressor in a variety of tumors [113–117]. The expression of hsa_circ_0001649 in GC was markedly decreased than that in their paired non-tumorous tissues [108]. Importantly, compared with the preoperative plasma samples, the expression level of hsa_circ_0001649 was up-regulated after surgery, revealing that hsa_circ_0001649 might be a postoperative follow-up marker in GC patients. Furthermore, clinical analysis showed that hsa_circ_0001649 level was observably relevant to tumor differentiation. What’s more, the expression of hsa_circ_0001895 was significantly downregulated in five GC cell lines (AGS, BGC-823, HGC-27, MGC-803, and SGC-7901) than that in normal gastric epithelial cells [109]. Compared with healthy tissues, hsa_circ_0001895 also showed lower level in GC tissues and gastric precancerous lesions. The expression level of hsa_circ_0001895 was correlated with GC cell differentiation, Borrmann type, and CEA level. These are some basic studies in which circRNAs are regarded as promising biomarkers for the diagnosis and prognosis of GC. However, more work is required before circRNAs are used in clinics.

### Therapeutic potential of circRNAs in GC

#### Potential anti-cancer effects of circRNAs

More and more evidences indicate that circRNAs play an important role in the proliferation and progression of GC cells and can serve as suppressors to regulate the development of GC.

As a tumor suppressor, miR-7 has been demonstrated to directly target and inhibit key oncogenic molecules involved in malignancies progression. Actually, lower expression of miR-7 was associated with the regulation of proliferation, invasion and metastasis in cancer [47, 49, 55]. CiRS-7 binds to miR-7 and inhibits its activity, which is involved in the development of various tumours [45, 55, 118]. The ciRS-7/miR-7 regulatory axis is intimately linked to the proliferation of gastrointestinal tumour cells [119]. Pan et al. found that overexpression of ciRS-7 could interfere with miR-7-mediated tumour suppression and enhance the PTEN/PI3K/AKT pathway, thereby promoting the proliferation of GC cells [83].

CircHIPK3 is a circRNA with multiple miRNA binding sites [73]. CircHIPK3 binds to a variety of GC-associated miRNAs by serving as sponges, such as miR-124 and miR-29b. The up-regulated expression of circHIPK3 in GC negatively regulates the expression of miR-124 and miR-29b, which affects the proliferation of GC cells and is closely related to the T stage and Ming’s classification of GC. In addition, the ability of circPDSS1 to promote the GC cell cycle and inhibit apoptosis by sponging miR-186-5p makes it a promising therapeutic target for GC [84].

A downregulated circRNA in GC tissues and cells, circFAT1, was identified by Fang et al. through analysing two sets of independent microarray datasets [71]. CircFAT1 acted as a sponge of miR-548 g and binded to the YBX1 protein to inhibit gastric tumour growth. Therefore, circFAT1 might serve as a potent suppressor and biomarker for the treatment and diagnosis of GC. Wang et al. identified several circRNAs and proved their functions in gastric carcinogenesis. CircNF1, which was up-regulated in GC tissues and cells, promoted cellular proliferation via sponging miR-16, thereby suppressing the effect of its downstream target genes like MAP7 and AKT3 [85].

Additionally, Liu and colleagues reported that circYAP1 was downregulated in GC, and acted as a tumor suppressor by sponging miR-367-5p to further inhibit the downstream target p27, thus inhibiting GC cell proliferation and invasion [86]. Zhong’s group reported that hsa_circ_0000993 could inhibit proliferation, migration and invasion of GC cells by sponging miR-214-5p, which was consistent with the results of clinical analysis that the patients with low level of miR-214-5p showed a better survival rate [87]. Another new research found that circ-ZFR was a low expressed circRNA that sponged miR-107/miR-130a in GC, further inhibited proliferation and facilitated apoptosis through regulating of PTEN signalling [88].

Besides, in a study of Cai’s group, the authors found a high expressed circRNA, circHECTD1, was related to lymph node metastasis and cancer stage in GC [89]. Knocking down of circHECTD1 caused inhibition of glutaminolysis, proliferation, migration, and invasion in GC cells. Further study demonstrated that circHECTD1 directly targeted miR-1256 and thereby increased the expression level of USP5, which is an activator of β-catenin/c-Myc signalling pathway. The circHECTD1/miR-1256/USP5 axis might provide enlightenments and potential targets for GC therapy. Given the important role of circRNAs in the life processes related to the development and progression of GC and the regulatory networks involved, it is reasonable to believe that circRNAs can serve as new targets for combating cancer in the future.

#### GC therapy targeting circRNAs

CircRNAs are involved in several cytological events in the development of GC, such as cell proliferation, migration, invasion and apoptosis, which provides possibilities for circRNAs as potential therapeutic targets for GC. Liu et al. transcribed a linear RNA molecule containing a miR-21 binding site in vitro, and circularisation was achieved by T4 RNA ligase after dephosphorylation and phosphorylation [120]. They concluded that this artificial circRNA could be synthesised in vitro, which inhibited cancer cell proliferation and the activity of miR-21 on downstream targets. Synthetic circRNA revealed a simple, effective and convenient strategy to intervene in specific miRNA functions in vitro and could be developed into a potential treatment for GC in the future.

Importantly, the study of Zhang et al. revealed that ciSR-133 was associated with the browning of white adipose tissue in patients with GC [90]. GC extracellular bodies transmitted ciRS-133 to preadipocytes and promoted the differentiation of preadipocytes into brown adipocytes by activating PRDM16 and inhibiting miR-133. The down-regulation of ciRS-133 attenuated cancer cachexia in mice with xenograft tumours and reduced oxygen consumption and heat production.

In addition, Huang et al. proved that the up-regulation of circAKT3 is associated with tumour invasiveness in GC patients who have received cisplatin (CDDP) treatment [91]. The expression of circAKT3 in CDDP-resistant GC samples was higher compared to CDDP-sensitive samples. Mechanism study demonstrated that circAKT3 promoted DNA damage repair and suppressed apoptosis by sponging miR-198 and increasing the expression of downstream target PIK3R1. This finding promised a new vision of therapy for CDDP-resistant GC patients.

### Methods for circRNA identification and representation

#### Identification tools for circRNAs

A variety of methods have been developed to study the structures and functions of circRNAs. RNA-seq and microarray technology are widely used for the identification of new circRNA species and the quantification of circRNA expression. Chen et al. distinguished 15,623 circRNA candidates from ribosomal RNA-depleted RNA samples [75]. Boeckel et al. identified 7388 circRNAs in the ribosomal RNA-depleted RNA of human umbilical vein endothelial cells, among which cZNF292 has the effect of promoting angiogenesis [121]. In addition, high-throughput sequencing was performed in fission yeast [122], protist [123] and plants [124].

Whether the identification of circRNAs is accurate and comprehensive depends on the rigour and reliability of the algorithm. Currently, a number of bioinformatics algorithms for circRNA prediction and identification have been developed. CircRNA_finder, find_circ, CIRCexplorer, CIRI and MapSplice are the five most commonly used circRNA prediction methods. Hansen et al. used these five tools to perform circRNA prediction on four sets of RNA-seq data; the number of detected circRNAs ranged from 1532 to 4067 [125]. The data obtained using different analytical tools varies: CIRI has the highest sensitivity and can detect the most circRNAs; Mapsplice has the highest accuracy and the lowest false positive rate; CircRNA_finder has the fastest analysis speed. It was proved that the combined analysis of any two methods could significantly reduce the false positive rate. The false positive rate is the lowest when the five methods are combined.

RNase R is a member of the *E. coli* RNR superfamily and can cleave RNA in the 3′ to 5′ direction [126, 127]. RNase R can degrade most linear RNAs, while circRNAs with closed loop structures are retained. Genome-wide high-throughput sequencing of circRNAs requires processing by RNase R to remove linear RNAs so as to increase the relative concentration of circRNAs [128]. Then, two approaches can be used to verify circRNAs: reverse transcription-polymerase chain reaction (RT-PCR) and Northern blot.

In RT-PCR, RNase R-digested RNA was reverse transcribed by random primers to get cDNA, and further amplified by divergent primers and convergent primers respectively, followed by separation of the amplified product in an agarose gel [16]. Both divergent and astringent primers can amplify the product to produce a band in the RNase R (−) group. While in the RNase R (+) group, the divergent primer generates a band but the convergent primer cannot. These results demonstrate the true presence of circRNAs and resistance to RNase R digestion.

Northern blot is a method of identifying circRNAs using specific probes [129]. In the RNase R (+) group, only the band of the circRNA could be seen due to the digestion of the linear mRNA. In the RNase R (−) group, both linear mRNA and circRNA could be visualised. In addition, fluorescence in situ hybridisation (FISH) is a powerful method for analysing the subcellular localisation of circRNAs [130]. Fluorescently labelled probes are designed to complement the sequence near the circRNA junction site. Cells were observed under high-resolution microscopy after the disposal of fixation, permeabilisation and probe hybridisation.

Furthermore, dual luciferase reporter assay [131], RNA immunoprecipitation (RIP) [132] and RNA pulldown-high-resolution mass spectrometry [133] can be used to reveal the interaction between circRNAs and miRNAs as well as circRNAs and RBPs.

#### Online resources

In recent years, an increasing number of circRNA research tools have been developed and improved functional analysis generated. This section presents online databases that are currently used for circRNA identification, prediction, localisation, characterisation and functional analysis, as well as tools for investigating the interaction of circRNAs with targets. Some of the online databases accessible for the study of circRNAs are shown in Table 3.

**Table 3:** Database for circRNA research

Database	URL	Annotation	Ref.
circBase	http://www.circbase.org/	A circRNA database contains circRNA information from multiple species.	[38]
circRNABase	http://starbase.sysu.edu.cn/mirCircRNA.php	Views the predicted miRNA-circRNA interactions by scanning circRNA sequences overlapping with CLIP-Seq peak.	[134]
Circ2Traits	http://gyanxet-beta.com/circdb/	A database of circular RNAs that may be associated with disease and traits. Predicts the interaction between miRNAs and genes, lncRNAs, and circRNAs.	[135]
CircInteractome	http://circinteractome.nia.nih.gov/	Predicts binding sites of proteins to circRNAs. Predicts potential binding sites for miRNAs-circRNAs interaction.	[136]
CircNet	http://circnet.mbc.nctu.edu.tw/	New circRNA predictions and genome annotations performed using 464 RNA sequencing data.	[137]
Deepbase	http://deepbase.sysu.edu.cn/	Collects 18,000 small RNAs, 36,0000 lncRNAs and 100,000 circRNA genes (human, murine, fruit flies, nematodes, etc.).	[138]
circRNADb	http://reprod.njmu.edu.cn/circrnadb	The first database that summarises circRNAs that encode proteins. A total of 32,914 human exon circRNAs were collected.	[139]
CIRCpedia	http://www.picb.ac.cn/rnomics/circpedia/	Constructs by using CIRCexplorer2 for bioinformatic predictive analysis of circRNAs in tissues and cell lines.	[140]
CSCD	http://gb.whu.edu.cn/CSCD	Collects circRNAs from tumour cell lines and normal cells from ENCODE. Predicts cellular localisation of circRNAs, MRE, RBP and variable splicing of related genes.	[141]
circlncRNAnet	http://app.cgu.edu.tw/circlnc/	Allows for a personalised analysis of lncRNAs, including lncRNA expression maps, gene function enrichment analysis maps, RBP-RNA network maps and miRNA network maps.	[142]
circRNA disease	http://cgga.org.cn:9091/circRNADisease/	CircRNA records include circRNA basic information, related disease information, circBase links and PubMed links.	[143]

## Conclusion and perspective

GC is a multi-step and multi-factor comprehensive disease, and the specific pathogenesis is still not fully understood. CircRNAs were once thought to be mistakes in the process of RNA splicing and are now considered to be an emerging class of RNA molecules with important functions. These abundant and stable RNAs are considered to be miRNA molecule sponges, protein baits, RNA splicing regulators, parental gene transcriptional regulators, potential protein translation templates, biomarkers and tumour suppressors. The function of circRNAs involves a variety of physiological and pathological processes.

In recent years, many studies have demonstrated the application of circRNAs in the clinical treatment of cancer. As described in this review, circRNAs are involved in numerous biological processes in the development and progression of GC, such as cell proliferation, migration, invasion, apoptosis, cachexia and drug resistance. As an important biomarker for the diagnosis and prognosis of GC, circRNAs are potential targets for the treatment of GC.

In conclusion, circRNAs provide a new perspective on the diagnosis and treatment of GC. However, compared with the coding RNAs, lncRNAs and miRNAs, our current understanding of circRNAs is still insufficient, and we are still far from applying circRNAs to clinical practice.

Here are some suggestions for future circRNA research. First, although there have been many studies, the exact mechanism by which circRNAs are involved in the development of cancer remains to be further studied. Second, current research on circRNAs in cancer mainly uses tumour cells and tumour tissue samples. Non-invasive samples (blood, urine, saliva, etc.) should be promoted for research and detection. Third, future studies could consider circRNAs as potential cancer therapeutic targets, how to transport circRNAs to relevant sites for effective long-term effects, and how to achieve no immune rejection (an urgent problem to be solved). Fourth, since the ultimate goal of circRNA research is to apply circRNAs for clinical treatment of human diseases safely, a large number of clinical studies and experiments are required.

We believe that through the development of techniques for identifying and screening novel circRNAs, as well as the improvement of online databases, circRNAs will one day be widely used for the diagnosis, treatment and prognosis monitoring of GC, thus bringing great progress to GC therapy.

## Data Availability

Not applicable.

## Abbreviations

ADAR1: Adenosine deaminase acting on RNA
AUC: Area under the curve
CA19–9: Carbohydrate antigen 19–9
CDDP: Cisplatin
CDK2: Cyclin-dependent kinase 2
CEA: Carcinoembryonic antigen
ceRNA: Competitive endogenous RNA
circRNA: Circular RNA
ciRNA: Circular intronic RNA
ecRNA: Exonic circRNA
elciRNA: Exon-intron circRNA
EMT: Epithelial-mesenchymal transition
FISH: Fluorescence in situ hybridization
GC: Gastric cancer
HDAg: Hepatitis D virus antigen
IRES: Internal ribosome entry site
lncRNA: Long non-coding RNA
m6A: N-methyladensine
MBL: Muscleblind
miRNA: MicroRNA
MRE: MiRNA response element
ncRNA: Non-coding RNA
OS: Osteosarcoma
p21: Cyclin-dependent kinase inhibitor 1
QKI: Quaking
RBP: RNA binding protein
RIP: RNA immunoprecipitation
RISC: RNA-induced silencing complex
RNase R: Ribonuclease R
RNA-seq: RNA sequencing
RT-PCR: Reverse transcription-polymerase chain reaction
snRNP: Small nuclear ribonucleic protein
SRY: Sex determining region Y
